# An immunohistochemical study of thioredoxin domain-containing 5 expression in gastric adenocarcinoma

**DOI:** 10.3892/ol.2014.2832

**Published:** 2014-12-29

**Authors:** ZHIMING WU, LIN ZHANG, NAN LI, LINA SHA, KUNPENG ZHANG

**Affiliations:** 1 Department of Gastroenterology and Hepatology, The 309 Hospital of People’s Liberation Army, Beijing 100091, P.R. China; 2 Hebei North University, Zhangjiakou, Hebei 073000, P.R. China

**Keywords:** gastric cancer, thioredoxin domain-containing 5 gene, immunohistochemistry, prognosis

## Abstract

Thioredoxin domain-containing 5 (TXNDC5) is overexpressed in a number of human carcinomas. However, the involvement of TXNDC5 in gastric adenocarcinoma remains unclear. In the present study, the immunohistochemical expression and clinicopathological significance of TXNDC5 in gastric adenocarcinoma was investigated. The immunohistochemical expression of TXNDC5 was detected in 54 gastric adenocarcinoma specimens, and the correlation between TXNDC5 and the clinicopathological features was investigated. Of the 54 gastric adenocarcinoma specimens, 30 samples (55.6%) exhibited high TXNDC5 expression. In the adenocarcinoma specimens exhibiting high TXNDC5 expression, the proportion of poorly-differentiated adenocarcinomas was significantly higher than that in specimens exhibiting low TXNDC5 expression (P<0.05). Lymph node metastasis and the depth of tumor invasion in the specimens exhibiting high TXNDC5 expression were significantly higher than that in specimens exhibiting low TXNDC5 expression (P<0.05). The results of a survival analysis revealed that the prognosis of patients exhibiting high TXNDC5 expression was significantly poorer than that of patients exhibiting low TXNDC5 expression (P<0.05). Therefore, the expression of TXNDC5 may correlate with the differentiation, invasion and metastasis of gastric adenocarcinoma. Thus, TXNDC5 may be a tumor-enhancing gene that is involved in gastric cancer.

## Introduction

Gastric cancer is the most common malignant tumor worldwide (1,2). The most common pathological type of gastric cancer is adenocarcinoma. Gastric adenocarcinoma is the most common type of carcinoma in China. As a complex multifactorial process, gastric carcinogenesis is believed to involve numerous genes and their products (3–5). In our previous study, comparative proteome analysis revealed that the expression of thioredoxin domain-containing 5 (TXNDC5) was significantly upregulated in certain precancerous lesions of gastric cancer, such as varioliform gastritis (VG), compared with the peripheral normal gastric mucous membrane (6). Thus, we hypothesized that upregulation of the TXNDC5 gene may lead to increased proliferation, as well as enhanced invasive activity, indicating a potential oncogene.

TXNDC5 was first identified in 2003 using two-dimensional gel electrophoresis analysis of the endoplasmic reticula of hepatic tissue (7). TXNDC5, which is a protein disulfide isomerase-like protein, was found to be highly expressed in endothelial cells. Furthermore, Sullivan *et al* (7) reported that TXNDC5 protects endothelial cells from stress-induced apoptosis. An increasing number of studies have revealed that the upregulation of TXNDC5 is found in tumors of the cervix, uterus and lungs (7–9). According to these studies, the TXNDC5 gene is hypothesized to be a tumor-enhancing gene, however, studies regarding the involvement of the TXNDC5 in gastric cancer remain limited. Furthermore, the association between TXNDC5 expression and clinicopathological factors in gastric adenocarcinoma remains unclear. In the present study, the expression of TXNDC5 in gastric adenocarcinoma was investigated by immunohistochemistry and the clinicopathological significance of TXNDC5 gene expression was investigated.

## Materials and methods

### Materials

Polyclonal goat anti-human TXNDC5 antibody (1:1,000) was purchased from Cell Signaling Technology, Inc., (Danvers, MA, USA). Rabbit anti-goat horseradish peroxidase (HRP)-conjugated antibody (dilution 1:200) was purchased from Beijing Zhongshan Golden Bridge Biotechnology Co., Ltd., (Beijing, China).

### Sample collection

Samples were obtained from 86 patients with gastric cancer treated at the 309 Hospital of the People’s Liberation Army (Beijing, China) between 1995 and 2008. The patients were diagnosed by gastroscopy and biopsy, and the results were confirmed by gastric resection, three-field lymph node dissection and reconstruction of the digestive tract. No patients received preoperative therapy. All samples were obtained during surgery. Four tissue sections of the tumor and normal mucosa were obtained from each patient and the tissue was embedded in paraffin for future use. The tissue sections were classified as well- or poorly-differentiated according to pathological diagnosis. Immunohistochemical analysis of the 86 patient samples revealed that 32 samples exhibited negative TXNDC5 staining, whereas 54 exhibited positive immunohistochemical staining (positive rate, 62.8%). Of the 54 patients, 37 were male and 17 were female, with a mean age of 60.6 years (mean ± standard deviation, 60.6±13.1 years). Written informed consent was obtained from all patients. This study was approved by the Ethics Committee of the 309 Hospital of the People’s Liberation Army. The clinicopathological stages of the tumors were assessed according to the International Union Against Cancer tumor-node-metastasis classification (10). Follow-up of the patients was performed after surgery until mortality. The mean duration of the follow-up period was two years and eight months (range, 1 month and 7 days to 4 years and 8 months).

### Immunohistochemical assay of TXNDC5

Tissue sections (5 μm) were cut from paraffin-embedded tissue blocks, placed on slides precoated with silane and incubated for 20 min at 60°C.

Subsequent to being washed in xylene and a graded series of ethanol to remove the paraffin, the sections were washed with phosphate-buffered saline (PBS) for 10 min. The sections were then treated with 2% bovine serum albumin (BSA; Beijing Huamaike Biotechnology Co., Ltd., Beijing, China) and 0.1% Triton X-100 (Shanghai Suolaibao Biotechnology Co., Ltd., Shanghai, China) in PBS (Shanghai Kexing Biotechnology Co., Ltd., Shanghai, China) for 1 h at room temperature. Next, the sections were treated with 3% H_2_O_2_ for 10 min to block endogenous peroxidase activity. Subsequent to being washed in PBS for 10 min, polyclonal goat anti-TXNDC5 antibody was applied for 1 h. After three washes with PBS, the biotin-labeled rabbit anti-goat HRP secondary antibody was added for 20 min. Next, the sections were washed in PBS for 10 min, then 3,3′-diaminobenzidine was applied as the chromogen. Finally, the sections were counterstained with Harris’ hematoxylin for 3 min and coverslips were applied with a xylene-based mounting medium. For the semiquantification of TXNDC5, the immunostaining was analyzed based on the criteria that were presented by Kase *et al* (11) and Nozoe *et al* (12). The TXNDC5-labeling index (showing the positive proportion of TXNDC5) was expressed as the percentage of the number of TXNDC5-labeled cells divided by the total number of cells examined under a microscope (20× objective). The average value of 10 fields was calculated.

High TXNDC5 expression was represented by ≥50% of the carcinoma cells in a specimen exhibiting positivity for TXNDC5. Specimens with <50% of the carcinoma cells exhibiting positivity for TXNDC5 were considered to exhibit low TXNDC5 expression.

### Reverse transcription-polymerase chain reaction (RT-PCR) assay of TXNDC5

Total RNA (1,400 ng/μl) was extracted by homogenization using TRIzol reagent (Invitrogen Life Technologies, Carlsbad, CA, USA). cDNA was synthesized in a 20-μl reverse transcription reaction system using 5 μg RNA. TXNDC5 was amplified and β2-MG was used as the internal control, in a DNA thermal cycler (PerkinElmer, Inc., Waltham, MA, USA) using equal amounts of cDNA as a template. The PCR products were separated by 1.5% agarose gel electrophoresis, then scanned and analyzed using an ImageMaster VDS System (GE Healthcare Life Sciences, Uppsala, Sweden).

### Statistical analysis

Student’s t-test and the χ^2^ test were used to compare the data. A survival analysis was performed using the Life Tables method. All statistical analyses were performed using SPSS version 11.0 (SPSS, Inc., Chicago, IL, USA). P<0.05 was considered to indicate a statistically significant difference.

## Results

**Figure 1:**
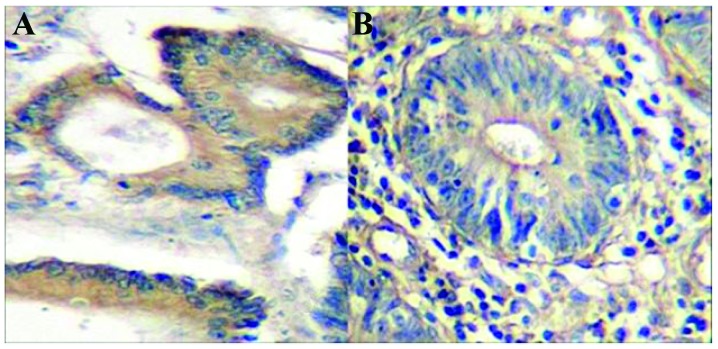
Immunohistochemical analysis. (A) Brown positive signals were predominantly distributed in the cytoplasm of the cancer cells in the samples with high TXNDC expression (SABC stain; magnification, ×200). (B) Weaker brown positive signals and fewer positive cells were identified in the samples with low TXNDC expression (SABC stain; magnification, ×200). TXNDC5, thioredoxin domain-containing 5; SABC, streptavidin-biotin complex.

The results of the immunohistochemical assay revealed brown staining, representing positive signals, predominantly distributed in the cytoplasm of the cancer cells, with a small proportion of in the nuclei and cell membrane (Fig. 1).

The results of the statistical analysis of the association between TXNDC5 expression and the clinicopathological characteristics are shown in Table I. A total of 30 patients (55.6%) exhibited high TXNDC5 expression, whereas 24 patients (44.4%) exhibited low levels of expression. No significant differences were identified between the patients with high TXNDC5 expression and those with low expression with regard to gender or age. The proportion of primary tumors located in the cardia was significantly higher in the specimens with high TXNDC5 expression (P<0.05). Furthermore, the proportion of poorly-differentiated adenocarcinomas was significantly higher in the specimens with high TXNDC5 expression compared with the specimens exhibiting low TXNDC5 expression (P<0.05). The proportion of lymph node metastases and the depth of the tumors in the specimens with high TXNDC5 expression was significantly higher than that in the low TXNDC5 expression group (P<0.05). No significant differences were identified between the high and low expression groups with regard to vascular invasion. Furthermore, a significant difference was identified between the two groups with regard to tumor stage.

**Figure 2:**
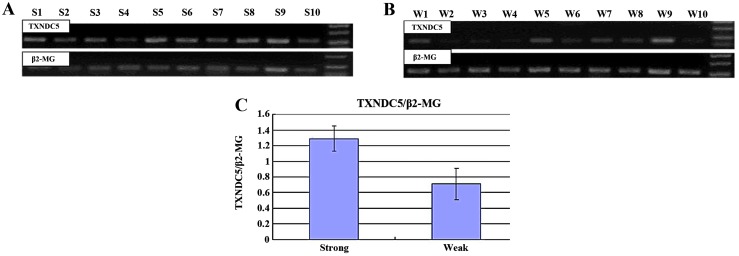
RT-PCR assay of TXNDC5. (A) RT-PCR assays for representative samples of the high TXNDC5 expression group. Higher expression of TXNDC5 in gastric cancer tissues with strong immunohistochemical staining was confirmed at the RNA level. (B) RT-PCR assays for representative samples of the low TXNDC5 expression group. Lower expression of TXNDC5 in gastric cancer tissues with weak immunohistochemical staining was confirmed at the RNA level. (C) The bands were quantified by densitometry scanning. The relative quantification was calculated as the ratio of TXNDC5 expression to β2-MG expression as shown above. RT-PCR, reverse transcription polymerase chain reaction; TXNDC5, thioredoxin domain-containing 5.

Using semiquantitative RT-PCR, 476-bp fragments of TXNDC5 and 876-bp control fragments of β2-MG were amplified (Fig. 2A and B). The mean ratios of the absorbency of the TXNDC5 band normalized to the control band were 1.29±0.16 and 0.71±0.20 in the high and low expression groups, respectively. This difference was significant when analyzed using Student’s t-test (P<0.05; Fig. 2C). The results also identified significant differences in TXNDC5 expression at the mRNA level between the high and low expression groups.

**Figure 3:**
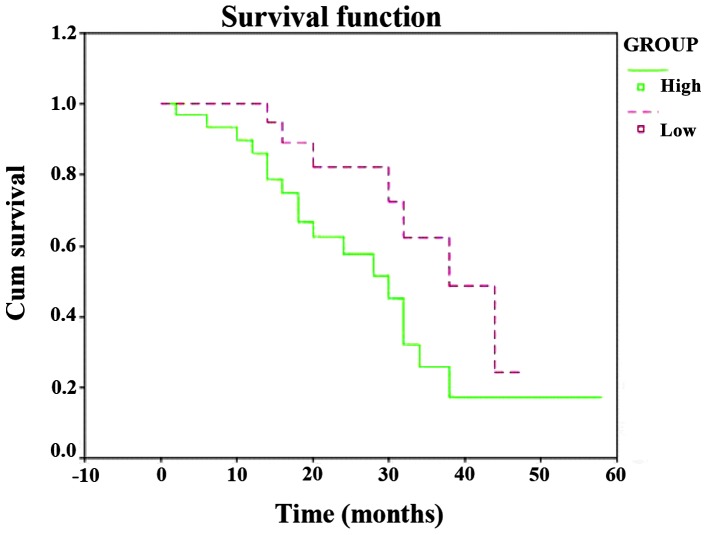
Survival curves of gastric cancer patients exhibiting different levels of TXNDC5 expression. The median survival time of the group with high TXNDC5 expression was significantly lower than that of the group with low TXNDC5 expression (P=0.035). TXNDC5, thioredoxin domain-containing 5.

The results of the survival analysis are shown in Fig. 3. The 10-, 20-, 30- and 40-month survival rates of the patients with high TXNDC5 expression were 83.3, 46.7, 23.3 and 6.7%, respectively, and those of the patients with low TXNDC5 expression were 87.5, 50.0, 29.2 and 8.3%, respectively. The median survival time for the group with high TXNDC5 expression was 28.47 months and that of the group with a weak TXNDC5 expression was 37.77 months. The prognosis of the patients with high TXNDC5 expression was significantly worse than those with low TXNDC5 expression (P=0.035).

## Discussion

At present, gastric cancer remains a common disease worldwide, with a poor prognosis and low survival rate. VG is a unique type of gastritis and a major precursor lesion of gastric cancer. Previous studies have demonstrated that VG is a significant step in gastric carcinogenesis (13–15). In a recent study, we detected a difference in protein expression between VG and the morphologically normal mucosa tissues near the lesions using the proteomic analysis (6,16). Results of these studies revealed that TXNDC5 could be a novel protein in those differentially-expressed proteins and that the cell cycle, cell death or proliferation-modifying processes may be involved in the precancerous change. However, the molecular mechanism behind the action of TXNDC5 is poorly understood.

Located on chromosome 6p24, the TXNDC5 gene encodes a protein-disulfide isomerase. To date, studies regarding the TXNDC5 gene are limited. It has been reported that the expression of the TXNDC5 gene is upregulated in a number of carcinoma tissues compared with normal tissues (17–20). However, few studies have investigated the expression of this gene in gastric cancer tissues. In the current study, the expression of the TXNDC5 gene in gastric adenocarcinoma tissues was detected using immunohistochemistry, and the association between TXNDC5 expression and clinicopathological features was analyzed. The results indicated that the TXNDC5 gene was expressed in gastric adenocarcinoma and that the positive signals were predominantly located in the cytoplasm of the tumor cells. The upregulated expression of TXNDC5 may correlate with poorly-differentiated adenocarcinoma, lymph node metastasis and deeper tumor invasion. Nissom *et al* (18) demonstrated that poorly-differentiated hepatocellular carcinoma (HCC) exhibits upregulated TXNDC5 expression, but that the level is unchanged in well-differentiated HCC, and thus, it was hypothesized that the gene is involved in tumor progression. In the present study, the proportion of the primary tumors located in the cardia was significantly higher in specimens exhibiting high TXNDC5 expression levels compared with those exhibiting low expression levels. The may be due to the fact that tumors located in cardia are often considered to be poorly-differentiated. A previous study indicated that the TXNDC5 gene may affect certain biological characteristics of cancer cells, promoting the growth and proliferation of tumor cells, or preventing their apoptosis (7). However, the exact molecular mechanism of this remains unclear and thus, further study to investigate the functional role of the gene *in vitro* and *in vivo* is required.

In conclusion, the immunohistochemical expression of TXNDC5 may correlate with poor differentiation of the tumors and with a poor prognosis in gastric cancer patients.

## Figures and Tables

**Table I:** Association between TXNDC5 expression and clinicopathological characteristics.

Clinicopathological parameter	Low TXNDC5 expression	High TXNDC5 expression	P-value
Age, years	59.3±10.4	61.6±15.2	P>0.05
Gender, n (%)			
Male	17 (70.8)	20 (66.7)	P>0.05
Female	7 (29.2)	10 (33.3)	P>0.05
Body weight, kg	66±12.3	69±19.4	P>0.05
Height, m	1.69±0.21	1.71±0.16	P>0.05
Primary tumor diameter, cm	4.3±2.6^a^	6.2±1.8^a^	P<0.05
Depth of invasion of primary tumor, n (%)			
T_0_	0 (0.0)	0 (0.0)	P>0.05
T_1_	3 (12.5)	3 (10.0)	P>0.05
T_2_	9 (37.5)^a^	5 (16.7)	P<0.05
T_3_	7 (29.6)	12 (40.0)^a^	P<0.05
T_4_	5 (20.8)	10 (33.3)^a^	P<0.05
Location of the primary tumor, n (%)			
Cardia	4 (16.7)	9 (30.0)^a^	P<0.05
Gastric body	6 (25.0)	8 (26.7)	P>0.05
Gastric antrum	9 (37.5)	10 (33.3)	P>0.05
Pylorus	5 (20.8)^a^	3 (10.0)	P<0.05
Lymph node metastasis, n (%)	7 (29.2)	16 (53.3)^a^	P<0.05
Vascular invasion, n (%)	10 (41.7)	14 (46.7)	P>0.05
Pathological type, n (%)			
Well-differentiated adenocarcinoma	9 (37.5)	10 (33.3)	P>0.05
Poorly-differentiated adenocarcinoma	7 (29.2)	13 (43.3)^a^	P<0.05
Signet ring cell carcinoma	3 (12.5)	3 (10.0)	P>0.05
Mucinous adenocarcinoma	4 (16.7)	3 (10.0)	P>0.05
Undifferentiated carcinoma	1 (4.2)	1 (3.3)	P>0.05

Quantitative data are expressed as the mean ± standard deviation.

a Significantly higher percentage.

TXNDC5, thioredoxin domain-containing 5.
